# Unveiling the Intracellular Survival Gene Kit of Trypanosomatid Parasites

**DOI:** 10.1371/journal.ppat.1004399

**Published:** 2014-12-04

**Authors:** Daniella Castanheira Bartholomeu, Rita Marcia Cardoso de Paiva, Tiago A. O. Mendes, Wanderson D. DaRocha, Santuza M. R. Teixeira

**Affiliations:** 1 Departamento de Parasitologia, Universidade Federal de Minas Gerais, Belo Horizonte, Minas Gerais, Brazil; 2 Departamento de Bioquímica e Imunologia, Universidade Federal de Minas Gerais, Belo Horizonte, Minas Gerais, Brazil; 3 Departamento de Bioquímica e Biologia Molecular, Universidade Federal do Paraná, Curitiba, Parana, Brazil; Boston College, United States of America

## Abstract

Trypanosomatids are unicellular protozoans of medical and economical relevance since they are the etiologic agents of infectious diseases in humans as well as livestock. Whereas *Trypanosoma cruzi* and different species of *Leishmania* are obligate intracellular parasites, *Trypanosoma brucei* and other trypanosomatids develop extracellularly throughout their entire life cycle. After their genomes have been sequenced, various comparative genomic studies aimed at identifying sequences involved with host cell invasion and intracellular survival have been described. However, for only a handful of genes, most of them present exclusively in the *T. cruzi* or *Leishmania* genomes, has there been any experimental evidence associating them with intracellular parasitism. With the increasing number of published complete genome sequences of members of the trypanosomatid family, including not only different *Trypanosoma* and *Leishmania* strains and subspecies but also trypanosomatids that do not infect humans or other mammals, we may now be able to contemplate a slightly better picture regarding the specific set of parasite factors that defines each organism's mode of living and the associated disease phenotypes. Here, we review the studies concerning *T. cruzi* and *Leishmania* genes that have been implicated with cell invasion and intracellular parasitism and also summarize the wealth of new information regarding the mode of living of intracellular parasites that is resulting from comparative genome studies that are based on increasingly larger trypanosomatid genome datasets.

## Trypanosomatids: Distinct Life Cycles, but Not So Divergent Genomes

Trypanosomatids (order Kinetoplastida) constitute a group of early-branching unicellular eukaryotes, which includes several human parasites responsible for diseases that affect over 20 million people and cause countless infections in other mammals, primarily in developing countries. Chagas disease (American trypanosomiasis), caused by *T. cruzi*—sleeping sickness or Human African Trypanosomiasis (HAT)—caused by *Trypanosoma brucei gambiensis*, *Trypanosoma brucei rhodesiensis*, and different forms of leishmaniases, caused by various species of *Leishmania*, are categorized amongst the most important neglected diseases causing approximately 150,000 deaths annually. In addition, *Trypanosoma vivax*, *Trypanosoma congolense*, and *Trypanosoma brucei* are pathogenic species in livestock and responsible for considerable production losses in South American and African countries (www.who.int/topics/tropical_diseases/en/). In spite of this large burden and the increasing efforts made by a relatively small group of researchers, no suitable vaccines for these diseases are available and the treatment is limited to a few drugs that have several undesirable side effects.

Kinetoplastids are protozoans characterized by the presence of a single branched mitochondrion containing a unique mitochondrial DNA structure known as kinetoplast [1]. Being early-branching eukaryotes, these organisms possess many peculiar characteristics, some of them reminiscent of their prokaryotic ancestors. Among the unusual features are genomic organization consisting of large, unidirectional gene clusters that are polycistronically transcribed [2], RNA polymerase I-mediated transcription of protein coding genes [3], RNA trans-splicing coupled to poly(A) addition [4], [5], and extensive RNA editing of mitochondrial mRNAs [6]. Besides their medical relevance, these unusual characteristics have driven the focus of intense research which may, hopefully, result in the development of new forms of treatment and disease prevention.

**Figure 1:**
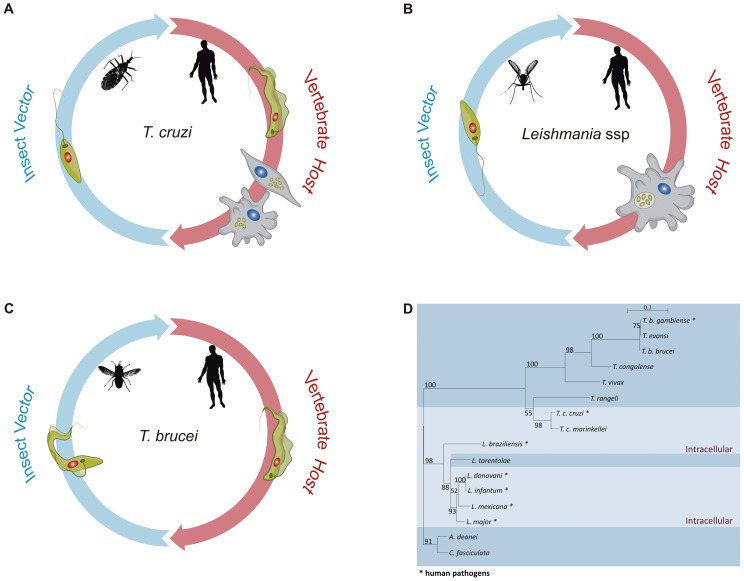
The distinct life cycles of tritryp parasites. Panels A–C show the life cycles of *T. cruzi*, *Leishmania* spp, and *T. brucei*, respectively. In each panel, some of the parasite stages present in their insect vectors, *T. cruzi* epimastigotes, *Leishmania* promastigotes, and *T. brucei* procyclic forms, are shown on the left. Different sand fly species of the genera *Lutzomyia* and *Phlebotomus* are vectors for Leishmania. *Triatoma infestans* and *Rhodnius prolixus* are the most important vector species in the transmission of *T. cruzi* to man, whereas different species of *Glossina*, also known as tse-tse fly, are vectors of African trypanosomes. Leishmania and *T. brucei* parasites move from the fly midgut up to the mouthparts before being inoculated into the human host as metacyclic, infective forms. Although Leishmania promastigotes achieve their journey in sand flies by being regurgitated from the stomodeal valve to the mouthparts, *T. brucei* epimastigotes do not stay in the mouthparts, as they have to first migrate from the proventriculus to the salivary glands where they develop into metacyclic forms and are expelled with the insect saliva. In contrast, *T. cruzi* infective metacyclic trypomastigotes develop in the hindgut of the triatomine bug and, after being excreted with the insect feces, gain access to the mammalian host bloodstream through skin wounds or the mucous membranes. On the right side of each panel, parasite forms present in the mammalian host, *T. cruzi* trypomastigotes, and intracellular amastigotes, *Leishmania* intracellular amastigotes, and *T. brucei* bloodstream forms are shown. Whereas *Leishmania* promastigotes are internalized by host phagocytes and reside into the phagolysosome, *T. cruzi* trypomastigotes actively invade a variety of nonphagocytic cells and are able to escape from the phagocytic vacuole and multiply in the host cell cytoplasm. Although distinct developmental forms of *T. brucei* are found in the mammalian host, namely stumpy and slender trypomastigotes, they remain extracellular during the entire parasite life cycle and were represented here as bloodstream trypomastigotes. Panel D shows a phylogenetic analysis inferred from glycosomal glyceraldehyde 3-phosphate dehydrogenase (gapdh) nucleotide sequences from 16 trypanosomatid species, with the species that have an intracellular stage shown with a light blue color. The maximum likelihood tree was constructed with 849 nt (80% of gapdh coding sequences), using SeaView v.04 and rooted at the *Crithidia fasciculata*/*A. deanei* clade, with the bootstrap values for 1,000 replicates shown in the major basal nodes.

The life cycles of all *Trypanosoma* and *Leishmania* species that cause human diseases depend on insect vectors (Figure 1). *Leishmania* spp proliferates as promastigotes in the midgut of phlebotomine sand flies and is transmitted to several species of mammals as metacyclic promastigotes when the fly takes a blood meal. In the mammalian host, *Leishmania major* is phagocytosed by macrophages and, once in the phagolysossome, metacyclic forms are converted into amastigotes, which multiply numerous times before being released during cell lysis [7]. *T. cruzi* replicates as epimastigotes in the midgut of different species of reduviid bugs and develops into infective metacyclic trypomastigotes once they reach the rectum and are excreted with the insect feces. In contrast to *L. major*, which multiplies only inside mammalian host macrophages, *T. cruzi* trypomastigotes invade essentially any nucleated cell type by a mechanism involving either lysosomal recruitment at the parasite invasion site or invagination of the plasma membrane followed by intracellular fusion with lysosomes. Also in contrast to *Leishmania* spp, *T. cruzi* trypomastigotes are able to escape from the phagolysosome into the cytosol where they differentiate into amastigotes [8]. After several rounds of cell division, amastigotes differentiate again into trypomastigotes that are released from the infected cell. *T. cruzi* amastigotes prematurely released from heavily infected cells can be also taken up and replicate within neighboring cells (See [9] and [10] for recent reviews on *T. cruzi* and *Leishmania* internalization processes). Different from *T. cruzi* and *Leishmania* spp, *T. brucei* develops extracellularly throughout its entire life cycle. It multiplies as procyclic forms in the intestinal tract of the tsetse fly before being transformed into infective metacyclic forms in the salivary glands. After being injected into the host during a blood meal, *T. brucei* proliferates in the bloodstream [11]. Therefore, unlike *T. cruzi* and *Leishmania*, which are able to hide inside mammalian cells, *T. brucei* needs to cope with the direct exposure to a strong antibody response in the host. To achieve this, it acquired a sophisticated immune evasion protocol, known as variant surface glycoprotein (VSG) switching. VSGs are encoded by a large family of *T. brucei*-specific genes whose monoallelic expression in bloodstream trypomastigotes results in a tightly packed surface coat of variant glycoproteins that shields other invariant surface proteins from the attack by the host immune system, allowing the parasite to multiply indefinitely in the bloodstream [12]. Thus, the lack of VSG genes in *T. cruzi* and *Leishmania* correlates with their ability to invade mammalian cells and by doing so, they do not need to cope with the continuous attack by the host humoral immune response.

The complete genome sequences of *T. brucei* [13], *T. cruzi* [14], and *L. major* [15], known as the tritryp genomes, represent a landmark in the study of these parasites. In contrast to the *T. brucei* and *L. major* genomes, whose 25 and 33 Mb sequences were assembled into 11 and 36 chromosomes, respectively, the much larger CL Brener *T. cruzi* genome (55 Mb haploid genome) has not been fully assembled, due to its repetitive and hybrid nature and the high level of allelic polymorphism. Although additional efforts resulted in the assembly of 41 pairs of chromosomes [16]. the exact chromosome number in *T. cruzi* is still not known. Despite a divergence period estimated between 200 to 500 million years, a conserved proteomic core derived from about 6,200 genes and a surprisingly large conservation of gene synteny were found between the tritryp genomes [17]. In the first comparative analysis published together with the description of the tritryp genomes, the authors remarked that the intracellular parasites, *L. major* and *T. cruzi*, appear to share slightly more genes than do *T. brucei* and *T. cruzi* and considerably more than do *L. major* and *T. brucei*. Whereas a total of 482 genes are shared between *L. major* and *T. cruzi*, only 74 common genes are present in the genomes of *T. brucei* and *L. major* [17]. Multigene families, retroelements, and structural RNAs often present in regions of synteny breaks were considered important elements that have shaped each parasite genome accordingly to their life cycles. Besides VSG genes, genes encoding elements of the RNA interference (RNAi) machinery were found exclusively in the *T. brucei* genome [17], [18]. Since a major role of RNAi silencing pathways is to down-regulate transcripts derived from transposons and repeats to maintain genome integrity, the absence of an RNAi machinery in *L. major* seems compatible with their lack of active transposons as well as with the mechanism of gene amplification involving the production of extrachromosomal circular DNA elements [19]. Like in other organisms, RNAi has been largely used as a tool for functional studies in *T. brucei* [20]. In contrast, due to the absence of functional RNAi machinery in *T. cruzi* and *L. major* [21], [19], functional genomics has evolved at a much slower pace in these two parasites. However, with the recent discovery that RNAi is functional in *Leishmania* (*Viannia*) *braziliensis* and other members of the Viannia subgenus [19], gene function studies in this *Leishmania* subgroup may start to catch up. Even more importantly, the conservation of the RNAi pathway in only a few *Leishmania* species may be correlated with the presence of dsRNA virus, known as Leishmania RNA Virus (LRV), with possible medical implications derived from recent data showing that infection by *Leishmania* isolates bearing LRVs results in metastatic forms of disease [19], [22].

## *T. cruzi* and *Leishmania* Genes Involved with Host Cell Invasion and Intracellular Multiplication

**Figure 2:**
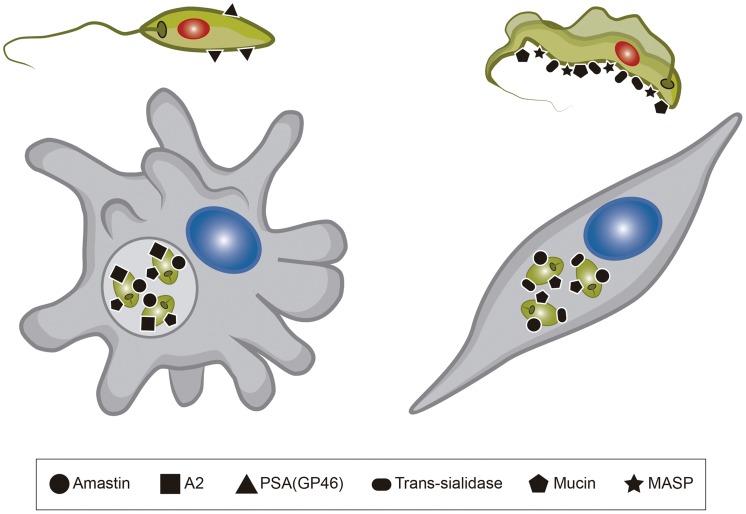
Surface proteins present in *Leishmania* and *T. cruzi*. The figure shows six different surface molecules known to be present in promastigote and amastigote forms of *Leishmania* (left) and trypomastigote and amastigote forms of *T. cruzi* (right). Each protein is represented by the symbols indicated below the figure.

Work from several groups has been dedicated to the characterization of genes known as virulence factors in *T. cruzi* and in different *Leishmania* species (Table 1) [23], [24]. Two groups of proteins present exclusively in *Leishmania*, the Promastigote Surface Proteins (PSAs), also known as the GP46 family, and the surface protein known as A2, both of which contain large amino acid repetitive domains, have been extensively characterized (Figure 2). PSAs are Leucine Rich Repeat (LRR)–containing surface proteins that bind to host cell macrophages and protect *Leishmania infantum* from complement-mediated lysis [25], [26]. The A2 genes encode a family of 42 to 100 kDa proteins made up almost entirely of 40 to 90 copies of a repetitive amino acid sequence, mainly expressed in amastigotes of *Leishmania donovani* and *L. infantum*, and that are known to be involved with parasite visceralization [27], [28]. Exogenous expression of A2 genes in *L. major*, which possesses only a truncated A2 pseudogene, enhanced the ability of *L. major*–infected cells to migrate out of the dermis and increase parasite survival in visceral organs [29]. Similarly, expression of A2 in *Leishmania tarentolae*, a lizard parasite which is nonpathogenic to mammals, resulted in increased *L. tarentolae* survival in mouse visceral organs [30].

**Table 1:** *T. cruzi* and *Leishmania* genes involved with host cell invasion and intracellular survival.

Organism	Gene Products	Protein Class	References
*T. cruzi*	Transialidase (GP85, GP83, Tc85, FL160, ASP, GP82, GP90)	Trans-sialidase superfamily	[36], [40], [65]–[67]
	GP35/50	Mucin protein	[68]
	GP63	Metallopeptidase	[69]
	Tc80	Serine protease	[70]
	Cruzipain (GP57/51)	Cysteine protease	[71]
	Amastin	Amastigote surface glycoprotein	[35]
	Oligopeptidase B	Serine peptidase	[72]
	LYT1	Lytic factor	[73]
	Tryparedoxin peroxidase	Antioxidant enzymes	[74]
*Leishmania*	GP63	metalloprotease	[75]
	Lipophosphoglycan Biosynthesis Enzymes	LPG biosynthesis	[76], [77]
	LIT1	Iron transporter	[78]
	LHRI	Heme transporter	[79]
	A2	Amastigote-specific protein	[29]
	ISPs	Inhibitor of serine peptidases	[80]
	Ascorbate peroxidase	Antioxidant enzymes	[81]

Amastin genes, which are also present in *T. cruzi*, are part of the group of trypanosomatid genes with up-regulated expression in amastigotes [31]. Initially characterized as *T. cruzi* and *Leishmania* amastigote specific genes [32], amastin genes have been identified in the genomes of other trypanosomatids, including the insect parasites, *Leptomonas seymouri*, *Angomonas deanei*, and *Strigomonas culicis* [33], [34]. Evidence indicating a role of this family of surface proteins related to intracellular survival and parasite dissemination within the mammalian host has been found recently in studies where amastin genes were overexpressed in the low infective *T. cruzi* G strain, which has reduced levels of amastin expression [35].

Mucins, Trans-sialidases (TSs), and Mucin Associated Surface Proteins (MASPs) are products of the three largest and highly heterogeneous gene families present in the *T. cruzi* genome [14]. TS catalyzes the transfer of sialic acid from sialylated donors present in the host cells to the terminal galactose residues of mucins present in the *T. cruzi* cell surface [36]. Whereas Mucin and MASP genes are exclusively found in *T. cruzi*, TS genes are also present in the genome of African trypanosomes, but not in *Leishmania* spp. However, in contrast to *T. cruzi* that has more than 1,400 copies of TS genes, the TS-like gene family has only nine members in *T. brucei* [37], 17 members in *T. congolense* [38], and about 100 members in *Trypanosoma rangeli* (E. Grisard, personal communication). Considerably less copies of TS are also present in *T. rangeli*, an insect parasite nonpathogenic for humans that does not have TS activity [39]. Evidence for the involvement of *T. cruzi* TS in the earlier steps of host cell invasion exit from the parasitophorous vacuole to the cytoplasm and the subsequent differentiation of trypomastigotes into amastigotes has been described by several groups [40], [41]. Thus, the massive expansion of the TS gene family in *T. cruzi* compared to *T. brucei*, its absence in *Leishmania* spp, and the lack of TS activity of the *T. rangeli* enzyme is likely to reflect not only the distinct niches these different trypanosomatids occupy within the infected host but also the distinct mechanisms of parasite internalization used by intracellular parasites. Also in agreement with the role of TS in *T. cruzi* is its large repertoire of genes encoding mucins, which form a thick glycocalyx barrier at the surface of different forms of the parasite [42]. Since mucins are the major acceptors of sialic acid, the absence of mucin genes in *T. brucei* [13] and the presence of much fewer members of mucin genes in *T. rangeli* [39] are also consistent with the incapability of these two *Trypanosoma* species to invade mammalian cells. Conversely, the ability of *T. cruzi* to invade and multiply in the cytoplasm of distinct host cell types has been also associated with its large repertoire of MASP genes, which, similar to mucin genes, is a highly polymorphic gene family [43], [44] that is absent in *T. brucei* and has a much smaller allele repertoire in *T. rangeli* [45].

## Comparative Genomics among Trypanosomatids As a Tool to Identify a Gene Set Specific for Intracellular Parasites

With the advent of next generation sequencing technologies, full genome analyses of an increasingly large number of members of the trypanosomatid family are being published, allowing us to dig deeper into the analysis of the genetic similarities and differences behind their distinct life styles. Surprisingly, comparative genome analyses of sequences from two other *Leishmania* species, *L. infantum* and *L. braziliensis*, with the *L. major* genome revealed not only a remarkable conservation in overall gene synteny, but also no more than 200 genes presenting a differential distribution between the three species [46]. Similar analyses that include sequences from other species of the *Viannia* complex, also known as New World *Leishmania*, *Leishmania mexicana* and *Leishmania amazonensis*, confirmed that there is little variation in the overall gene content and indicated that gene amplification as well as variation in chromosome number and ploidy constitute major sources of genomic variation across *Leishmania* species [47], [48]. Since human leishmaniasis is characterized by a highly diverse spectrum of clinical symptoms, these studies suggest that additional factors besides differences in gene content across *Leishmania* species are likely to play a role in determining disease phenotype. Likewise, in spite of the fact that the lizard parasite *L. tarentolae*, which belongs to a third subgenera, the Sauroleishmania, is nonpathogenic to humans and does not multiply intracellularly, comparative studies identified only 95 predicted coding sequences unique to *L. (S) tarentolae* [49]. Furthermore, also highlighting our scarce understanding of the biology of intracellular parasites, most of the genes that are present in pathogenic *Leishmania* species and absent in *L. tarentolae* encode hypothetical proteins or proteins with unknown function.

Comparative genomic analyses between two *T. cruzi* strains, CL Brener and Sylvio X-10 strain [50], which belong to two phylogenetically distinct groups as well as from *Trypanosoma cruzi marinkellei*, a bat-associated parasite of the subgenus Schizotrypanum [51], also revealed few differences in their genome content. Again, copy number variation within the large multigene families appears to be a major determinant of subspecies variation in *Trypanosoma*. Similar to mammals infected by *T. cruzi*, bats infected by *T. cruzi marinkellei* contain intracellular amastigotes in cardiac, skeletal, and stomach muscle cells. Therefore, differences found in the genomes of *T. cruzi*, *T. cruzi marinkellei*, and the various *T. brucei* subspecies, such as the absence of MASP and mucin genes in *T. brucei*, can be associated with the lack of capacity of *T. brucei* to invade host cells. On the other hand, the observations that all *Trypanosoma* species, including *T. cruzi*, *T. cruzi marinkellei*, and *T. brucei*, have TS genes suggest that TS plays a role in the biology of trypanosomatid parasites regarding not only host cell invasion and intracellular survival but also parasite survival in the insect or in the mammalian bloodstream. Indeed, in GPI-anchored defective *T. brucei*, lack of surface TS strongly affects parasite survival in the insect midgut [52].

Genome studies of trypanosomatids other than *Trypanosoma* and *Leishmania* such as the recent sequence analysis of the genomes of two monoxenic insect parasites, *A. deanei* and *S. culicis*, again revealed strikingly conserved features when compared to pathogenic trypanosomatids, such as gene families encoding amastin and cysteine proteases. As expected, no sequences homologous to VSG, mucin-like glycoproteins, and TS genes were found [34]. Besides helping understand the distinct life cycles of these organisms, the relevance of this study relies on the fact that these trypanosomatids bear endosymbiotic bacteria and are considered excellent models for evolutionary studies, specifically how a host protozoan coevolved with an intracellular bacterium in a mutualistic relationship.

Another recent genome-wide study based on the complete genome sequences of 27 protozoans, 17 of them obligate intracellular parasites, six of them exclusively extracellular, and four free-living protists, showed that the predicted proteome of intracellular parasites have a higher content of repetitive sequences compared to extracellular parasites and free-living protists [53]. Therefore, this study suggests that the ability to invade host cells may have shaped the expansion and maintenance of amino acid repeats in the proteome of intracellular parasites. Indeed, tandemly repeated amino acid sequences are characteristic of many surface proteins of other intracellular protozoan parasites such as *Plasmodium* spp, that have been implicated with binding to host receptors and immune-evasion mechanisms [54]. Similarly, several *Leishmania* spp and *T. cruzi* proteins containing repeated amino acid motifs have been described as targets of B cell immune response, and a bias towards the expression of these proteins in the amastigote stage further suggests their involvement with intracellular parasitism [55], [56].

**Figure 3:**
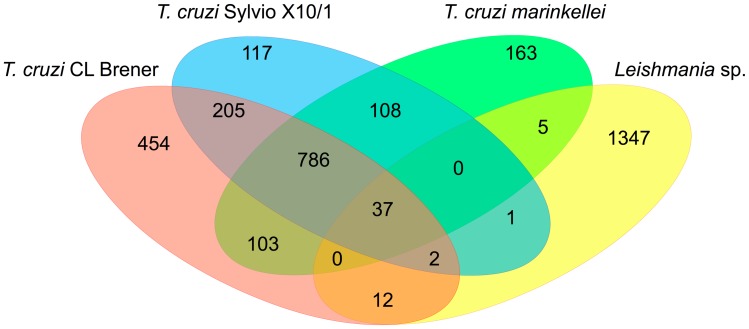
Common genes present exclusively in intracellular parasites. After identifying orthologous proteins, by performing an all-versus-all alignment between the amino acid sequences, the results of the pairwise alignments were used as input to the OrthoMCL software V1.4 with its default parameters. Specific OrthoMCL clusters of intracellular and extracellular/apathogenic trypanosomatids and functional enrichment analysis based in genome annotation were performed using in-house PERL scripts.

Genome mining of the Trypanosomatid sequence databases can still provide new sets of valuable information about *T. cruzi* and *Leishmania* genes that may be part of their intracellular survival gene kit. We analyzed the predicted protein sequences from 15 trypanosomatids for which the genome sequences are available and that were divided into two groups according to their ability to invade and survive inside mammalian host cells. As shown in Table S1, the first group is formed by species that have an intracellular stage and the second group includes trypanosomatids that are either nonpathogenic to mammals or that do not have an intracellular stage. From a total of 13,609 OrthoMCL clusters identified in the analyzed dataset, 3,340 were present only in intracellular parasites and approximately 1.0% of them (37 clusters) are shared between the two *T. cruzi* strains (CL Brener and Sylvio X-10), *T. cruzi marinkellei*, and six species of *Leishmania* (Figure 3). Over 60% of these clusters contain genes annotated as hypothetical proteins with no functional characterization (Table S2). Among the few proteins in this group that have been characterized as virulence factors, we identified kinases and phosphatases that are known to play important roles in host cell invasion in *T. cruzi* [57], [58] and differentiation and proliferation of amastigotes in *L. mexicana* [59], *L. donovani*, and *L. major* [60]. Also present in this group are mannosyltransferases and a putative fatty acid transporter. Mannan constitutes over 80% of the cellular carbohydrate content of *Leishmania* intracellular amastigotes [61], and the involvement of this sugar modification has been highlighted in studies with parasite mutants deficient in mannan metabolism which are unable to infect macrophages [62]. Increased fatty acid uptake is required in response to changes in the metabolism of intracellular parasites since a dramatic shift from carbohydrate- to lipid-dependent energy metabolism has been observed in *Leishmania* and *Trypanosoma* as an adaptation to the intracellular environment [63], [64]. Not surprisingly, the small number of common genes found between *Trypanosoma* and *Leishmania* is in agreement with the distinct mechanisms of host cell invasion adopted by these organisms as well as differences in the intracellular niches they occupy within their host cells.

By delivering more and more genomic sequence information, trypanosomatid genome databases will still provide a large portion of the foundations for our studies on the molecular basis of the intracellular life style of these parasites. Yet, experimental approaches based on genetic manipulation of these parasites are more necessary than ever to better characterize such a distinctive gene set. It is noteworthy the increasingly large difference in the pace with which these genomes are being explored, due to the faster advancement of genetic manipulation tools developed for *T. brucei*. For *T. cruzi* and *Leishmania*, efforts towards genome-wide experimental characterizations of sequences that can be associated with their intracellular life style are, therefore, much welcome.

## Supporting Information

Table S1 Proteome dataset used to identify orthologous sequences.
(DOCX)

Table S2 Conserved OrthoMCL clusters in intracellular trypanosomatids.
(XLSX)
