# Supplementary material for: Unveiling the Intracellular Survival Gene Kit of Trypanosomatid Parasites
Source: PLoS Pathog. 2014 Dec 4;10(12):e1004399. doi: 10.1371/journal.ppat.1004399 (PMC4256449; doi:10.1371/journal.ppat.1004399)
Supplement: Table S1 — Proteome dataset used to identify orthologous sequences. (DOCX) [file ppat.1004399.s001.docx]

Table 1 – Proteome dataset used to identification of orthologous

| **Organisms** | **Groups** | **Database** | **Genome version** | **Reference** |
| --- | --- | --- | --- | --- |
| *Angomonas deanei* | Extracellular/Apathogenic | GenBank | 1.0 | [1] |
| *Leishmania amazonensis* | Intracellular | GenBank | 1.0 | [2] |
| *Leishmania braziliensis* | Intracellular | TritrypDB | 6.0 | [3] |
| *Leishmania donovani* | Intracellular | TritrypDB | 6.0 | [4] |
| *Leishmania infantum* | Intracellular | TritrypDB | 6.0 | [3] |
| *Leishmania major* | Intracellular | TritrypDB | 6.0 | [5] |
| *Leishmania mexicana* | Intracellular | TritrypDB | 6.0 | [6] |
| *Strigomonas culicis* | Extracellular/Apathogenic | GenBank | 1.0 | [1] |
| *Trypanosoma brucei brucei* | Extracellular/Apathogenic | TritrypDB | 6.0 | [7] |
| *Trypanosoma brucei gambiensis* | Extracellular/Apathogenic | TritrypDB | 6.0 | [8] |
| *Trypanosoma cruzi strain CL Brener* | Intracellular | TritrypDB | 6.0 | [9] |
| *Trypanosoma cruzi strain Sylvio X10/1* | Intracellular | TritrypDB | 6.0 | [10] |
| *Trypanosoma cruzi marinkellei* | Intracellular | TritrypDB | 6.0 | [11] |
| *Trypanosoma congolense* | Extracellular/Apathogenic | TritrypDB | 6.0 | [12] |
| *Trypanosoma vivax* | Extracellular/Apathogenic | TritrypDB | 6.0 | [12] |
